# Etiology and Anatomical Level of Lower-Limb Amputations in Two Iranian Referral Hospitals: A Retrospective Record-Based Study

**DOI:** 10.3390/healthcare14203518

**Published:** 2026-10-19

**Authors:** Hossein Akbari Aghdam, Mehdi Motififard, Hasan Yazdekhasti, Fouad Rahimi, Mahsa Jamali, Mohammad Shahsavan

**Affiliations:** 1 Department of Orthopedic Surgery, Isfahan University of Medical Sciences, Isfahan 81746-73461, Iran; akbariaghdam@med.mui.ac.ir (H.A.A.);; 2 Department of Orthopedic Surgery, Faculty of Medicine, Kurdistan University of Medical Sciences, Sanandaj 66177-13446, Iran; 3 Joint Reconstruction Research Center, Tehran University of Medical Sciences, Tehran 14197-33141, Iran

**Keywords:** amputation, surgical, lower extremity, wounds and injuries, peripheral arterial disease

## Abstract

**Background**: Lower-limb amputation arises from heterogeneous pathways ranging from severe trauma to dysvascular disease and infection, yet comparative data across Iranian hospitals remain limited. We aimed to characterize demographic, etiologic, and anatomical patterns of lower-limb amputation and to evaluate between-hospital heterogeneity. **Methods**: This retrospective, two-hospital, record-based study included 229 eligible hospitalization records with lower-limb amputation at Al-Zahra and Kashani hospitals in Isfahan, Iran, from 13 April 2014 through 18 March 2020. Primary etiology was reconstructed from ordered diagnostic codes using a predefined hierarchical algorithm; external-cause codes were reviewed as supporting contextual information and to characterize documented traumatic mechanisms. Amputation level was derived from ICD-9-CM procedure codes. Analyses used Python 3.13.5 (NumPy, pandas, SciPy, and statsmodels). **Results**: Trauma was the leading etiology (49.3%), followed by dysvascular disease (28.8%) and infection (10.5%). Age differed strongly across etiologies (H = 93.71; Holm-adjusted *p* < 0.001; epsilon-squared = 0.398, 95% CI 0.307–0.509), with trauma-related hospitalizations occurring at younger ages. Toe (35.4%) and below-knee (34.5%) amputations were most frequent. Hospital was associated with both etiology and amputation level (both Holm-adjusted *p* < 0.001; bias-corrected Cramer’s V = 0.374 and 0.471, respectively). In an adjusted major-versus-minor model, older age and treatment at Al-Zahra Hospital were associated with major amputation, whereas female sex was not statistically significant. **Conclusions**: The hospitalizations showed marked descriptive differences by age, etiology, and hospital. Trauma-related hospitalizations occurred at younger ages, whereas dysvascular and infection-related hospitalizations occurred later in life. The substantial between-hospital differences indicate that pooled hospital-based estimates may obscure institutional case mix and should not be interpreted as population-level incidence.

## 1. Introduction

Lower-limb amputation represents one of the most consequential endpoints of severe limb pathology and is generally undertaken when preservation of a functional and viable extremity is no longer achievable. Although advances in vascular reconstruction, diabetic foot care, trauma management, antimicrobial therapy, and limb-salvage techniques have altered the indications for amputation, the procedure continues to carry a substantial burden of disability, loss of independence, impaired quality of life, and long-term health-care utilization. This burden is particularly pronounced after major lower-limb amputation, for which postoperative morbidity and mortality remain considerable, especially among patients with peripheral arterial disease, diabetes, renal dysfunction, and other systemic comorbidities [1,2,3,4].

The epidemiology of lower-limb amputation is highly heterogeneous and reflects the demographic, socioeconomic, and clinical characteristics of the population being studied. In older populations and settings with a high prevalence of diabetes and peripheral arterial disease, dysvascular disease accounts for a large proportion of amputations [1,5]. By contrast, trauma remains an important cause of limb loss, particularly among younger and economically active populations and in settings where road-traffic injuries, occupational trauma, and high-energy mechanisms are common. Global analyses of traumatic amputation have shown that, despite reductions in age-standardized rates over recent decades, the absolute numbers of incident cases, prevalent cases, and years lived with disability attributable to traumatic amputation have increased, with lower-limb loss contributing substantially to disability [6,7]. These differences indicate that the relative importance of trauma, dysvascular disease, infection, and malignancy cannot be reliably extrapolated from one health-care system or geographic region to another.

Regional epidemiology is particularly relevant in the Middle East and North Africa, where lower-limb amputation patterns are shaped by the simultaneous burden of diabetes and vascular disease, population aging, trauma, and substantial variation in access to preventive and specialist care. A recent analysis of Global Burden of Disease data demonstrated marked heterogeneity in lower-limb amputation trends across countries in the region and emphasized the continuing scarcity of detailed epidemiological data needed to inform locally appropriate prevention strategies [7]. Hospital-based estimates may also vary because the populations captured by individual institutions can differ substantially.

Available evidence from Iran includes an earlier single-hospital retrospective study from northwestern Iran in which trauma was the leading cause of lower-limb amputation, followed by vascular disease [8]. That study predominantly included major amputations and did not evaluate between-hospital variation. More recent Iranian studies have largely focused on selected high-risk populations, including patients with diabetic foot disease [9]. The present study therefore extends the available Iranian evidence by directly comparing two hospitals within the same city and by examining both minor and major amputations across a broader etiologic spectrum.

Accordingly, we aimed to characterize the demographic profile, primary etiologies, and anatomical levels of lower-limb amputation recorded at Al-Zahra and Kashani hospitals in Isfahan, Iran. We further examined associations with age and sex, quantified between-hospital differences in etiologic and anatomical case mix, and explored temporal variation within each hospital. Because the source data were hospital based and lacked a population denominator, the study was not designed to estimate population incidence or prevalence.

## 2. Materials and Methods

### 2.1. Study Design and Setting

This retrospective, two-hospital, record-based observational study analyzed hospitalization records documenting lower-limb amputation at Al-Zahra and Kashani hospitals in Isfahan, Iran. The observed admission dates ranged from 13 April 2014 to 18 March 2020; these dates do not establish the intended database extraction period or confirm complete capture of eligible inpatient amputations. The unit of analysis was the eligible hospitalization record rather than the unique patient or an independently verified amputation episode. The study protocol was approved by the Ethics Committee of Isfahan University of Medical Sciences on 11 July 2020 (protocol code IR.MUI.MED.REC.1399.292). Reporting followed relevant STROBE recommendations and, where applicable, the RECORD extension for routinely collected health data [10,11,12].

### 2.2. Study Population

The two available hospital source extracts contained 229 hospitalization records with an ICD-9-CM lower-limb amputation procedure code (84.10–84.18): 130 from Al-Zahra Hospital and 99 from Kashani Hospital. All extracted records were assessed for eligibility. Independent inpatient procedure totals or extraction audit logs were unavailable, so comprehensive capture of all eligible amputations at either hospital, including during 2015–2019, could not be verified. Eligibility required hospitalization within the observed study period, documentation of lower-limb amputation, and sufficient demographic, diagnostic, and procedural information for analysis. Consistent with the original protocol, records with more than 80% missing study information were considered ineligible. For this criterion, completeness was assessed across five core analytic domains: age, sex, admission date, diagnostic information, and procedure information. No hospitalization had more than 80% of these domains missing; therefore, no record was excluded on this basis. All 229 extracted hospitalizations entered the final analytic sample, and no a priori sample-size calculation was performed. The ascertainment process is summarized in Supplementary Figure S1.

The source extracts represented inpatient hospitalizations; outpatient-only procedures were not captured. A uniform patient identifier was not available across both hospitals and all study years; therefore, repeated admissions by the same individual could not be reliably linked. Consequently, staged, revision, or contralateral amputations occurring during separate hospitalizations may have contributed more than one observation. The analytic unit was therefore the hospitalization record rather than the confirmed unique patient or individual amputation episode. Possible within-patient dependence could not be modeled because patient-level linkage was unavailable.

### 2.3. Data Collection and Harmonization

The two hospital datasets were reviewed and harmonized into a common analytic structure before pooling. Variables retained for analysis were hospital, age, sex, admission date, final diagnostic codes, external-cause codes, and surgical procedure codes. Source admission dates were converted to Gregorian calendar dates for analysis and reporting. Personal identifiers, including names and hospital record numbers, were excluded from the de-identified analytic dataset.

Age, sex, and at least one lower-limb amputation procedure code were available for all 229 hospitalizations. The available coding information was insufficient to assign a defensible primary etiology in 14 hospitalizations (6.1%); these were retained as other/indeterminate rather than classified by assumption. Anatomical amputation level remained unspecified in one hospitalization (0.4%). No statistical imputation was performed. For trauma-related hospitalizations, external-cause codes were additionally summarized descriptively to characterize documented injury mechanisms.

### 2.4. Classification of Primary Amputation Etiology

The source datasets did not contain a standardized variable identifying the primary indication for amputation. Primary etiology was therefore reconstructed from the ordered diagnostic fields using a deterministic hierarchical algorithm. The principal or first recorded diagnosis was evaluated first. If it identified a specific etiologic process, that category was assigned. If the first diagnosis was nonspecific or nonetiologic, subsequent diagnoses were reviewed in their recorded order, and the first specific anatomically relevant etiologic code determined classification. Gangrene (R02) and lower-limb ulceration (L97) were treated as nonspecific manifestations: when a more specific etiologic code was present, that code took precedence; otherwise, these manifestations were classified as dysvascular. A specific lower-limb injury or injury sequela generally took precedence over a nonspecific manifestation, whereas an anatomically unrelated injury did not establish traumatic etiology.

Each hospitalization was assigned to one of six mutually exclusive categories: trauma-related, dysvascular, infection-related, malignancy, deformity, or other/indeterminate. Diabetes alone did not determine primary etiology; diabetes with peripheral circulatory complications or documented peripheral arterial occlusive or thromboembolic disease supported dysvascular classification. Osteomyelitis and documented postoperative, stump, or internal-device infection were classified as infection-related when they represented the principal or first specific etiologic diagnosis. External-cause codes were reviewed as supporting contextual information and to characterize traumatic mechanisms, but did not independently determine the etiologic category. Two hospitalizations were operationally categorized as dysvascular using the nonspecific I99 diagnosis without a separate limb-specific vascular or gangrene/ulcer code, and one was categorized as traumatic using the nonspecific multiple-injury code T07; the original clinical charts were unavailable to verify these assignments. These three records are therefore subject to greater etiologic uncertainty. Age, sex, hospital, and amputation level were not used to assign etiology. The complete observed diagnostic-code inventory, external-cause code inventory, exact hierarchy and precedence rules, and worked examples are provided in Supplementary Table S1. The revised classification used a deterministic rule set rather than independent duplicate clinical adjudication; inter-rater agreement was therefore not estimated, and validation against complete clinical charts was not possible because those records were unavailable for the revised analysis.

A review of the ordered diagnostic codes identified four dysvascular hospitalizations (all at Al-Zahra Hospital) assigned through the R02/L97 fallback rule without a more specific qualifying etiologic code. Four additional dysvascular assignments relied on I99: two without accompanying R02/L97 or a limb-specific vascular code and two accompanied by R02. One trauma-related assignment relied on T07. Because the original clinical charts were unavailable for verification, these nine code-dependent assignments were examined in nested conservative sensitivity analyses by reclassifying them as other/indeterminate rather than assuming an alternative diagnosis.

### 2.5. Classification of Amputation Level

Anatomical amputation level was determined from ICD-9-CM procedure codes and categorized as toe amputation (84.11), partial foot amputation (84.12), ankle-level amputation (84.13–84.14), below-knee or transtibial amputation (84.15), knee disarticulation (84.16), above-knee or transfemoral amputation (84.17), and hip disarticulation (84.18). Code 84.12 is a broad partial foot category; the available source records did not contain sufficient operative detail to distinguish reliably among specific partial foot levels such as transmetatarsal, Lisfranc, or Chopart procedures. Hospitalizations coded as 84.12 were therefore retained as partial foot amputations without further anatomical subclassification.

When more than one amputation-level code was recorded during the same hospitalization, the most proximal documented level was retained to represent the final extent of amputation during that admission. For code 84.10 (lower-limb amputation, not otherwise specified), a specific level was assigned only when accompanying diagnostic information explicitly identified the anatomical site; otherwise, the level remained unspecified. For secondary severity analyses, toe and partial foot amputations were classified as minor, whereas ankle-level or more proximal amputations were classified as major; the single hospitalization with an unspecified level was excluded.

### 2.6. Statistical Analysis

Age was summarized using mean and standard deviation (SD) together with median and interquartile range (IQR) for overall descriptive reporting. Its distribution was assessed graphically and using the Shapiro–Wilk test. Because age significantly departed from normality (W = 0.973, *p* < 0.001), subgroup age distributions were presented as median (IQR), and rank-based nonparametric methods were used for inferential comparisons. Categorical variables were summarized as frequencies and percentages, with Wilson score 95% confidence intervals (CIs) for principal proportions. All inferential analyses were conducted at the hospitalization level and treated hospitalization records as independent observations because patient-level linkage was unavailable.

Age distributions across primary etiologic categories and anatomical amputation levels were compared using the Kruskal–Wallis test. Epsilon-squared was calculated as ε^2^ = (H − k + 1)/(n − k), where H is the Kruskal–Wallis statistic, k is the number of groups, and n is the number of observations. Following a significant omnibus test, pairwise etiologic comparisons were performed using Dunn’s test with tie correction, with Holm adjustment across all pairwise comparisons. Rank-biserial correlation was reported as the pairwise effect-size measure.

Associations between categorical variables were evaluated using a Fisher–Freeman–Halton Monte Carlo procedure for R x C contingency tables. The conditional probability of the observed table under fixed row and column margins was used for two-sided probability ordering. For each analysis, 200,000 random tables with the observed marginal totals were generated using the Patefield algorithm implemented in scipy.stats.random_table, and the Monte Carlo *p* value was calculated as (b + 1)/(B + 1), where b is the number of simulated tables with conditional probability less than or equal to that of the observed table and B is the number of resamples. Bias-corrected Cramér’s V was used to quantify categorical associations.

Ninety-five percent CIs for epsilon-squared, rank-biserial correlations, and bias-corrected Cramer’s V were obtained using 10,000 nonparametric percentile bootstrap resamples. For epsilon-squared and rank-biserial correlations, resampling was performed within comparison groups to preserve group sizes; for Cramer’s V, hospitalization records were resampled as paired observations. Because no single primary inferential hypothesis had been prespecified, the nine original inferential analyses were treated as one exploratory family and their *p* values were additionally adjusted using the Holm procedure. Raw and Holm-adjusted *p* values are presented in the Section 3. Reviewer-requested hospital-stratified, sensitivity, multivariable, and temporal analyses were considered secondary analyses and were interpreted primarily through effect estimates and CIs.

To assess potential confounding by hospital, age-by-etiology, age-by-sex, age-by-level, sex-by-etiology, and sex-by-level associations were examined separately within each hospital. Because several etiologic categories contained very small or zero cell counts after stratification, a six-category multivariable model for primary etiology was not fitted because the resulting estimates would be unstable. Anatomical levels were also dichotomized as minor (toe and partial foot) or major (ankle-level or more proximal), excluding the single unspecified record, and a multivariable binary logistic regression model was fitted with major versus minor amputation as the dependent variable and age, sex, and hospital as covariates. Sensitivity analyses excluded the 14 other/indeterminate hospitalizations from the hospital-by-etiology comparison and, separately, combined dysvascular and infection-related etiologies to assess the impact of possible overlap between these categories. Exploratory temporal comparisons were restricted to the available hospitalization records from Gregorian years 2015–2019 and performed separately within each hospital. The partial boundary years 2014 and 2020 were retained for descriptive reporting only; completeness of hospital ascertainment during 2015–2019 could not be verified. All tests were two-sided. Analyses were conducted using Python 3.13.5, NumPy 2.3.5, pandas 2.2.3, SciPy 1.17.0, and statsmodels 0.14.6.

The exploratory major-versus-minor logistic model included age, sex, and hospital to estimate associations conditional on the other variables in the model. Etiology was not included because it was reconstructed from diagnostic codes and several categories were sparse, potentially yielding unstable estimates in this sample. Consequently, the age and hospital estimates were not adjusted for etiologic case mix. Age was entered as a continuous variable per 10-year increment. Its assumed linear association with the log odds of major amputation was assessed using a likelihood-ratio comparison with a natural cubic spline specification (two additional degrees of freedom).

To assess dependence on nonspecific diagnostic codes, we repeated the etiologic analyses after reassigning (i) two I99-based dysvascular records without R02/L97 and one T07-based trauma record (three records); (ii) these three and four R02/L97 fallback records (seven records); and (iii) these seven and two additional I99-with-R02 records (nine records). All observations were retained (*n* = 229), with reassigned records categorized as other/indeterminate. We reassessed age-, sex-, hospital-, and amputation-level-by-etiology associations using the original tests and effect-size methods. These secondary analyses were not included in the original Holm-adjusted test family.

## 3. Results

### 3.1. Study Sample

A total of 229 eligible hospitalizations were included: 130 (56.8%) from Al-Zahra Hospital and 99 (43.2%) from Kashani Hospital. The mean age was 45.76 ± 21.58 years, and the median age was 44.0 years (IQR, 27.0–63.0; range, 2–96 years). Male sex was recorded in 198 hospitalizations (86.5%) and female sex in 31 (13.5%).

Median age was higher among female than male hospitalizations (61.0 years [IQR, 45.0–78.5] vs. 42.0 years [IQR, 27.0–60.8]; U = 1946.0; raw *p* = 0.0011; Holm-adjusted *p* = 0.0064; rank-biserial r = −0.366, 95% CI −0.572 to −0.142). Hospitalizations at Al-Zahra Hospital were also modestly older than those at Kashani Hospital (median, 48.5 vs. 39.0 years; U = 7483.0; raw and Holm-adjusted *p* = 0.0349; r = 0.163, 95% CI 0.009–0.307). Sex distribution did not differ significantly between hospitals (Fisher’s exact *p* = 0.437) (Table 1).

### 3.2. Primary Etiology of Amputation

Trauma-related conditions were the most frequent primary etiology, accounting for 113 hospitalizations (49.3%; 95% CI, 42.9–55.8%), followed by dysvascular disease in 66 (28.8%; 95% CI, 23.3–35.0%), infection in 24 (10.5%; 95% CI, 7.1–15.1%), deformity in 8 (3.5%; 95% CI, 1.8–6.7%), malignancy in 4 (1.7%; 95% CI, 0.7–4.4%), and other/indeterminate causes in 14 (6.1%; 95% CI, 3.7–10.0%). The hospital-specific distributions are summarized in Table 2. Diabetes codes (E10–E14) were present in 49 hospitalizations (21.4%); 46 of 66 dysvascular hospitalizations (69.7%) had a diabetes code.

**Figure 1:**
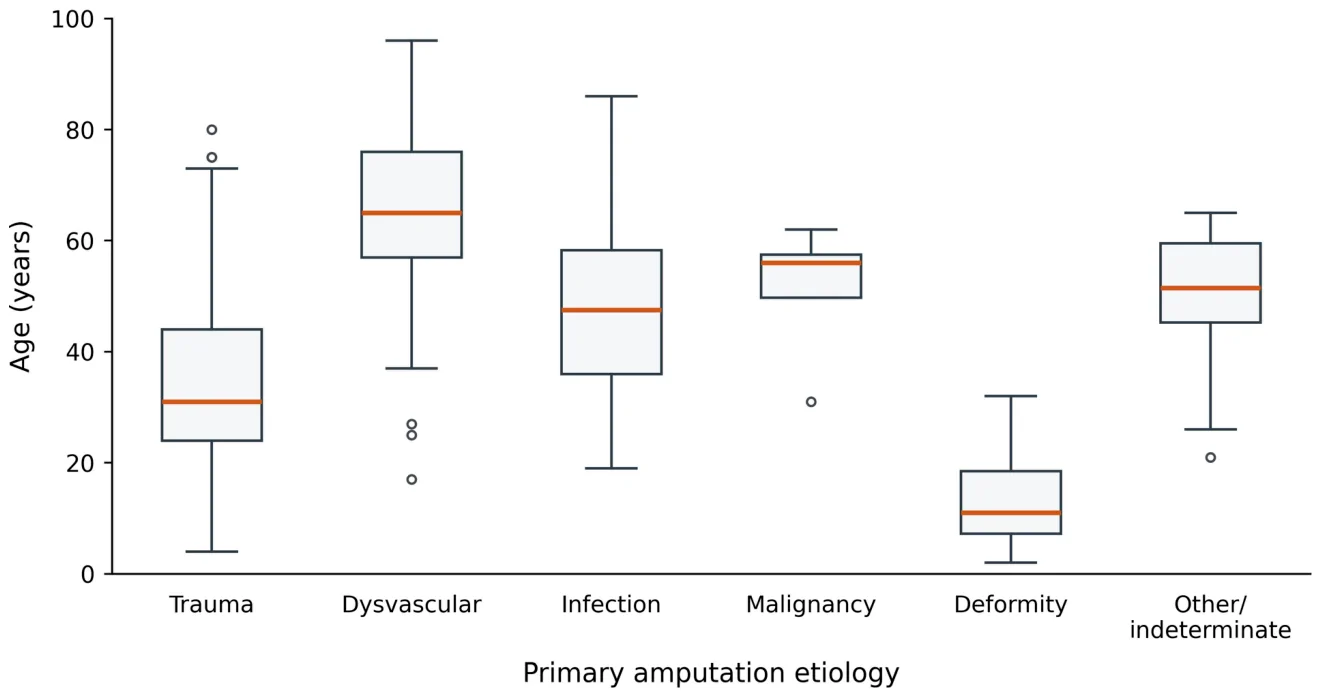
Age distribution according to primary amputation etiology after code-level reclassification. Boxes represent the interquartile range, horizontal lines denote medians, whiskers extend to observations within 1.5 times the interquartile range, and observations beyond the whiskers are displayed individually. The overall age distribution differed significantly across etiologic groups (Kruskal–Wallis H = 93.71; raw and Holm-adjusted *p* < 0.001; ε^2^ = 0.398, 95% CI 0.307–0.509).

Age differed strongly across etiologic categories (Kruskal–Wallis H = 93.71, df = 5; raw and Holm-adjusted *p* < 0.001; ε^2^ = 0.398, 95% CI 0.307–0.509). Median age was 31 years (IQR, 24–44) for trauma, 65 years (IQR, 57–76) for dysvascular disease, 47.5 years (IQR, 36.0–58.3) for infection, 56 years (IQR, 49.8–57.5) for malignancy, 11 years (IQR, 7.3–18.5) for deformity, and 51.5 years (IQR, 45.3–59.5) for other/indeterminate etiologies (Figure 1; Table 3).

Dunn-Holm post hoc testing showed that trauma-related hospitalizations occurred at younger ages than dysvascular (adjusted *p* < 0.001; rank-biserial r = −0.747, 95% CI −0.845 to −0.633) and infection-related hospitalizations (adjusted *p* = 0.0173; r = −0.489, 95% CI −0.672 to −0.285). Deformity-related hospitalizations occurred at younger ages than dysvascular (adjusted *p* < 0.001), infection-related (adjusted *p* < 0.001), and other/indeterminate hospitalizations (adjusted *p* = 0.0021). No other pairwise comparison remained significant after Holm correction (Supplementary Table S2).

**Figure 2:**
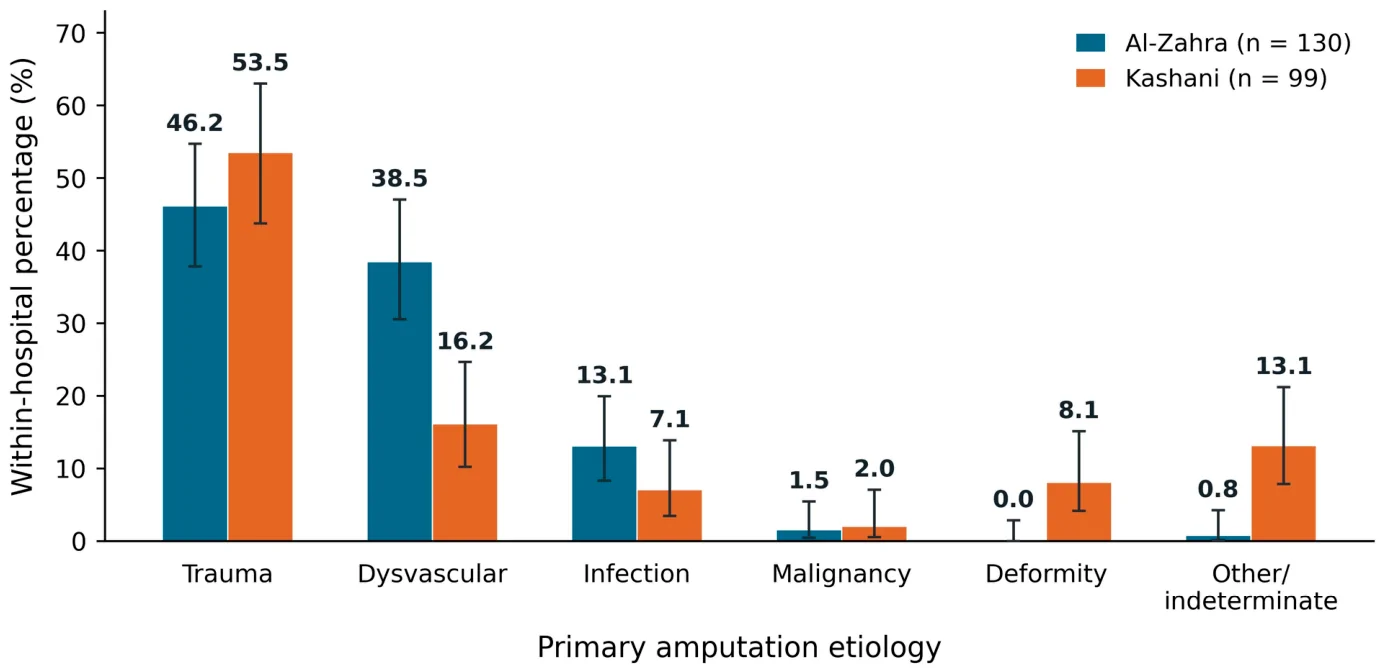
Distribution of primary amputation etiology by hospital after code-level reclassification. Bars represent within-hospital percentages, and error bars indicate 95% Wilson confidence intervals for the corresponding proportions (Al-Zahra, *n* = 130; Kashani, *n* = 99). Etiologic distribution differed significantly between the two hospitals (Fisher–Freeman–Halton Monte Carlo raw *p* < 0.001; Holm-adjusted *p* < 0.001; bias-corrected Cramér’s V = 0.374, 95% CI 0.285–0.486).

Primary etiology was associated with sex (Fisher–Freeman–Halton Monte Carlo raw *p* = 0.0065; Holm-adjusted *p* = 0.0195; bias-corrected Cramer’s V = 0.215, 95% CI 0.073–0.411). The etiologic distribution also differed substantially between hospitals (raw and Holm-adjusted *p* < 0.001; bias-corrected V = 0.374, 95% CI 0.285–0.486) (Figure 2; Table 2). This between-hospital association remained statistically significant after excluding all 14 other/indeterminate hospitalizations (Monte Carlo *p* < 0.001; bias-corrected V = 0.293, 95% CI 0.195–0.416). In a second sensitivity analysis combining dysvascular and infection-related hospitalizations into a single category, the principal findings were preserved: age remained strongly associated with etiology (H = 86.78, *p* < 0.001; epsilon-squared = 0.370, 95% CI 0.277–0.479), between-hospital etiologic distributions remained significantly different (Monte Carlo *p* < 0.001; bias-corrected V = 0.379, 95% CI 0.285–0.487), and the etiology-by-level association remained significant (Monte Carlo *p* = 0.0034; bias-corrected V = 0.200, 95% CI 0.103–0.441) (Supplementary Table S3).

Reassigning the three most uncertain I99/T07-based records to other/indeterminate did not eliminate the age–etiology or hospital–etiology associations (Supplementary Table S3). After reassignment of all seven records, including the four R02/L97 fallback assignments, age remained associated with etiology (H = 88.63, *p* < 0.001; ε^2^ = 0.375, 95% CI 0.288–0.486), as did hospital (Monte Carlo *p* = 0.00011; bias-corrected Cramér’s V = 0.281, 95% CI 0.189–0.411). The etiology–amputation level association, however, was no longer statistically significant (Monte Carlo *p* = 0.0522; V = 0.159, 95% CI 0.082–0.389). Reassigning nine records yielded similar findings, including an etiology–level *p* value of 0.0525 (Supplementary Table S3).

Among the 113 trauma-related hospitalizations, an external-cause code was available in 98 (86.7%). These codes permitted limited descriptive characterization of broad injury mechanisms: transport-related events were the most frequently documented specific mechanism (53 hospitalizations), followed by machinery-related injury (12) and being struck by a falling or projected object (11); firearm injury was documented in 2. An industrial or construction setting while working for income was explicitly coded in 12 trauma-related hospitalizations. These coded data did not provide sufficiently detailed information on injury circumstances, severity, or preventability for mechanism-specific causal inference.

### 3.3. Anatomical Level and Secondary Analyses

Toe and below-knee amputations were the most frequent anatomical levels, recorded in 81 hospitalizations (35.4%; 95% CI, 29.5–41.8%) and 79 (34.5%; 95% CI, 28.6–40.9%), respectively. Above-knee amputation occurred in 30 (13.1%), knee disarticulation in 17 (7.4%), partial foot amputation in 11 (4.8%), ankle-level amputation in 8 (3.5%), and hip disarticulation in 2 (0.9%); one hospitalization (0.4%) remained unspecified. The anatomical and grouped minor/major distributions by hospital are shown in Table 2.

Age differed across the seven specified levels (H = 18.71, df = 6; raw *p* = 0.0047; Holm-adjusted *p* = 0.0188; ε^2^ = 0.057, 95% CI 0.016–0.156). Amputation level was associated with sex (raw *p* = 0.0032; Holm-adjusted *p* = 0.0162; bias-corrected Cramér’s V = 0.260, 95% CI 0.117–0.457) and differed substantially by hospital (raw and Holm-adjusted *p* < 0.001; bias-corrected V = 0.471, 95% CI 0.374–0.592). Detailed statistics for the nine original exploratory comparisons, including raw and Holm-adjusted *p* values, are provided in Supplementary Table S4.

Primary etiology and anatomical level were also associated, although the effect was smaller (raw *p* = 0.0143; Holm-adjusted *p* = 0.0286; bias-corrected V = 0.170, 95% CI 0.098–0.394) (Table 4).

In hospital-stratified secondary analyses, age continued to differ strongly across etiologic categories within both Al-Zahra Hospital (H = 50.25, *p* < 0.001; epsilon-squared = 0.370, 95% CI 0.242–0.526) and Kashani Hospital (H = 41.39, *p* < 0.001; epsilon-squared = 0.391, 95% CI 0.244–0.578). The hospital-specific age-by-sex estimates were U = 600.0 (*p* = 0.0013; rank-biserial r = −0.455, 95% CI −0.694 to −0.190) at Al-Zahra and U = 388.5 (*p* = 0.290; r = −0.197, 95% CI −0.559 to 0.203) at Kashani. The age-by-level association was not statistically significant within either Al-Zahra Hospital (H = 12.57, *p* = 0.050; epsilon-squared = 0.053) or Kashani Hospital (H = 7.23, *p* = 0.204; epsilon-squared = 0.024). For sex-by-etiology, the hospital-specific estimates were bias-corrected Cramer’s V = 0.323 (95% CI 0.094–0.512; Monte Carlo *p* = 0.005) at Al-Zahra and V = 0.050 (95% CI 0.000–0.450; *p* = 0.224) at Kashani. The sex-by-level estimates were also imprecise in both hospitals (Supplementary Table S5).

Among 228 hospitalizations with a specified anatomical level, 92 (40.4%) were classified as minor and 136 (59.6%) as major (Table 2). In the exploratory major-versus-minor logistic model, older age (adjusted OR per 10 years = 1.20, 95% CI 1.04–1.39; *p* = 0.012) and hospitalization at Al-Zahra (adjusted OR = 4.25, 95% CI 2.37–7.60; *p* < 0.001) were associated with higher odds of major amputation. The association with female sex was imprecisely estimated and did not reach conventional statistical significance (adjusted OR = 2.65, 95% CI 0.96–7.29; *p* = 0.060; Table 5). A likelihood-ratio comparison with a natural cubic spline provided no evidence of departure from age linearity on the logit scale (χ^2^ = 0.104, df = 2, *p* = 0.949). These estimates were conditional on the included covariates but not adjusted for etiologic case mix.

### 3.4. Temporal Patterns

The earliest and latest observed admission dates among extracted records were 13 April 2014 and 18 March 2020; these do not establish the intended or fully covered database-extraction period. Annual counts were 26 in the partial year 2014, 33 in 2015, 64 in 2016, 48 in 2017, 24 in 2018, 32 in 2019, and 2 in the partial year 2020 (Table 6A). Exploratory temporal comparisons were restricted to 2015–2019 and performed separately within each hospital; uninterrupted, comprehensive capture of eligible inpatient amputations in those years could not be established.

Among the available Al-Zahra records, no statistically significant temporal variation was detected for age (H = 2.94, *p* = 0.569), primary etiology (Monte Carlo *p* = 0.910), or amputation level (*p* = 0.181). The corresponding comparisons among the available Kashani records were also non-significant for age (H = 3.13, *p* = 0.536), primary etiology (*p* = 0.486), and amputation level (*p* = 0.830). These secondary *p* values were not included in the Holm family used for the original inferential analyses. Hospital-stratified temporal results are reported in Table 6B.

## 4. Discussion

This study identified marked descriptive differences in age across etiologic categories among 229 lower-limb amputation hospitalizations: trauma-related hospitalizations occurred predominantly at younger ages, whereas dysvascular and infection-related hospitalizations occurred later in life. Trauma accounted for 49.3% of hospitalizations and dysvascular disease for 28.8%. Age differences across etiologies were large (epsilon-squared = 0.398), whereas age differences across anatomical levels were much smaller (epsilon-squared = 0.057). The age–etiology association remained strong within both hospitals, and the principal findings were also preserved when dysvascular and infection-related etiologies were combined in sensitivity analysis. These findings describe the observed hospital case mix and should not be interpreted as defining validated epidemiological or clinical profiles.

The prominence of trauma is directionally consistent with previous Iranian reports but should not be described as a direct replication. Rouhani and Mohajerzadeh reported trauma as the leading cause of lower-limb amputation in a single hospital in northwestern Iran, followed by vascular disease [8], while Mousavi et al. also identified trauma as a major cause in a nine-year Iranian series [13]. Those studies differed from the present analysis in setting, case ascertainment, etiologic definitions, and the representation of minor versus major amputations. The added value of the current study is therefore not the observation that trauma occurs frequently in Iran, but the direct comparison of two hospitals within the same city using a common code-based classification framework and inclusion of both minor and major procedures.

The age separation between etiologic groups was clinically marked. Trauma-related hospitalizations had a median age of 31 years, compared with 65 years for dysvascular disease and 47.5 years for infection. Earlier Iranian work likewise reported substantially younger ages for traumatic than vascular amputations [8], and a level-I trauma-center study by Dhillon et al. described post-traumatic amputation predominantly among younger adults [14]. By contrast, Iranian diabetic-foot cohorts have generally involved older populations [9]. External-cause codes permitted limited descriptive characterization of broad injury mechanisms in a subset of trauma-related hospitalizations. However, these codes did not provide sufficiently detailed information on injury circumstances, injury severity, occupational exposure, preventability, prehospital care, or referral delays to support mechanism-specific causal or preventive conclusions.

Sex-related findings require cautious interpretation. Male sex predominated overall, but the hospital-specific estimates for sex-related associations had substantial uncertainty (Supplementary Table S5). A statistically significant within-hospital association at Al-Zahra and a non-significant result at Kashani do not, by themselves, demonstrate a difference between hospitals; no formal interaction analysis was performed. In the exploratory major-versus-minor model, the association between female sex and major amputation was imprecisely estimated and did not reach conventional statistical significance (adjusted OR = 2.65, 95% CI 0.96–7.29; *p* = 0.060). The age–etiology association was observed within both hospitals but does not constitute a validated clinical classification. The model-based age and hospital estimates represent exploratory associations conditional on the other included covariates; because etiology was not included, they should not be interpreted as independent of etiologic case mix.

A major contribution of this study is the demonstration of substantial between-hospital heterogeneity, despite both hospitals being located in the same city. The hospital-by-etiology association remained significant after exclusion of the 14 other/indeterminate hospitalizations and after combining dysvascular and infection-related etiologies, reducing concern that uncertain or overlapping etiologic classification fully explained the finding. However, detailed information on referral pathways, catchment populations, service configuration, vascular capacity, and trauma transfer patterns was not available. The causes of the observed differences therefore cannot be determined from these data. Other hospital-based studies have likewise shown that case mix and inclusion criteria can strongly influence the apparent leading causes and levels of amputation [15,16,17,18,19], reinforcing the need for caution when pooling institutional data or extrapolating them to the general population.

In the available extracted records, no statistically significant within-hospital variation in age, etiology, or amputation level was detected across Gregorian years 2015–2019. Because completeness and consistency of extraction coverage over time could not be independently verified, these results cannot establish stable hospital-wide amputation patterns or population-level trends. Population-level studies from the Middle East and North Africa, Germany, Saskatchewan, and Belgium have reported temporal changes in major and minor amputation patterns [7,16,20,21]. The present study also cannot assess pandemic-related changes because only two included hospitalizations occurred in the partial year 2020 and observation ended on 18 March 2020.

The observed age and etiologic differences may provide context for future prevention and service-planning research, but they do not directly establish specific preventive strategies. In the broader literature, traumatic and dysvascular amputations are addressed through different prevention and limb-preservation approaches [15,22,23]. However, the present datasets did not contain sufficiently detailed information on trauma circumstances or severity, glycemic control, peripheral arterial disease severity, vascular interventions, referral delays, preventive care, smoking, or multidisciplinary limb-salvage pathways. Accordingly, recommendations concerning road-traffic or occupational injury prevention and dysvascular limb-preservation strategies should be regarded as contextual implications rather than conclusions derived directly from this study.

Conservative reclassification of uncertain I99, T07, and R02/L97 assignments did not eliminate the age–etiology or between-hospital associations. In contrast, the etiology–amputation level association was attenuated and no longer reached the conventional significance threshold when seven or nine code-dependent assignments were reassigned. This association should therefore be interpreted cautiously because it was sensitive to etiologic classification uncertainty.

Several limitations should be considered. The retrospective design depended on the completeness and accuracy of routinely coded diagnoses, external causes, and procedures, and primary etiology had to be reconstructed rather than prospectively recorded. The original extraction criteria and independent hospital procedure totals were unavailable; consequently, complete inpatient case ascertainment and consistency of coverage during 2015–2019 could not be verified. No independent duplicate clinical adjudication or validation against complete clinical charts was available; therefore, misclassification remains possible, especially in the I99-only and T07-coded hospitalizations and between complex dysvascular and infectious presentations, although the combined-category sensitivity analysis preserved the principal findings. The unit of analysis was the hospitalization, and repeated admissions by the same individual could not be reliably identified across the full dataset. Consequently, staged, revision, or contralateral procedures during separate admissions may have been represented as separate observations, within-patient clustering could not be modeled, and unrecognized correlation may have underestimated statistical uncertainty. Outpatient-only procedures were not captured. The two hospitals provide no population denominator, several etiologic and anatomical categories were small, and detailed information on trauma severity and circumstances, glycemic control, vascular status, referral delays, and prior preventive or limb-preservation care was unavailable. Code 84.12 did not permit reliable subclassification of the exact partial foot level. Although hospital-stratified and multivariable analyses were added where feasible, residual confounding remains possible. Future multicenter prospective or registry-based studies should use standardized etiologic definitions, patient-level linkage, detailed vascular and trauma variables, and population denominators to determine the generalizability and underlying causes of the patterns observed here.

## 5. Conclusions

Lower-limb amputation hospitalizations at the two studied hospitals showed substantial descriptive differences by age and etiology, with trauma-related hospitalizations occurring at younger ages and dysvascular and infection-related hospitalizations occurring later in life. Substantial differences in both etiology and anatomical level were also observed between hospitals, indicating that pooled hospital-based estimates can obscure important institutional case mix and should not be interpreted as population incidence. Multicenter prospective studies with standardized etiologic definitions, patient-level linkage, and more detailed clinical information are needed to determine the generalizability and drivers of these observed patterns.

## Figures and Tables

**Table 1:** Demographic characteristics of the analytic sample overall and by hospital.

Characteristic	Overall (N = 229)	Al-Zahra Hospital (*n* = 130)	Kashani Hospital (*n* = 99)	*p* Value
Age, mean ± SD, years	45.76 ± 21.58	48.42 ± 21.21	42.25 ± 21.67	
Age, median (IQR), years	44.0 (27.0–63.0)	48.5 (30.3–64.8)	39.0 (25.0–60.5)	0.0349
Male sex, *n* (%)	198 (86.5)	110 (84.6)	88 (88.9)	0.437
Female sex, *n* (%)	31 (13.5)	20 (15.4)	11 (11.1)	

SD, standard deviation; IQR, interquartile range. Age was compared between hospitals using the Mann–Whitney U test; sex distribution was compared using Fisher’s exact test. The unit of analysis is the eligible hospitalization.

**Table 2:** Distribution of primary amputation etiology and anatomical amputation level overall and by hospital.

Variable/Category	Overall *n* (%)	95% CI	Al-Zahra *n* (%)	Kashani *n* (%)
**Primary etiology**				
Trauma-related	113 (49.3)	42.9–55.8	60 (46.2)	53 (53.5)
Dysvascular	66 (28.8)	23.3–35.0	50 (38.5)	16 (16.2)
Infection-related	24 (10.5)	7.1–15.1	17 (13.1)	7 (7.1)
Malignancy	4 (1.7)	0.7–4.4	2 (1.5)	2 (2.0)
Deformity	8 (3.5)	1.8–6.7	0 (0.0)	8 (8.1)
Other/indeterminate	14 (6.1)	3.7–10.0	1 (0.8)	13 (13.1)
**Amputation level**				
Toe	81 (35.4)	29.5–41.8	23 (17.7)	58 (58.6)
Partial foot	11 (4.8)	2.7–8.4	10 (7.7)	1 (1.0)
Ankle	8 (3.5)	1.8–6.7	4 (3.1)	4 (4.0)
Below-knee	79 (34.5)	28.6–40.9	57 (43.8)	22 (22.2)
Knee disarticulation	17 (7.4)	4.7–11.6	7 (5.4)	10 (10.1)
Above-knee	30 (13.1)	9.3–18.1	27 (20.8)	3 (3.0)
Hip disarticulation	2 (0.9)	0.2–3.1	2 (1.5)	0 (0.0)
Unspecified	1 (0.4)	0.1–2.4	0 (0.0)	1 (1.0)
**Grouped amputation level (specified levels only)**				
Minor amputation	92 (40.4)	34.2–46.8	33 (25.4)	59 (60.2)
Major amputation	136 (59.6)	53.2–65.8	97 (74.6)	39 (39.8)

CI, confidence interval. Overall 95% CIs are Wilson score intervals. Partial foot denotes ICD-9-CM code 84.12; the operative records did not permit reliable identification of specific partial-foot levels. Minor amputation comprised toe and partial-foot amputations; major amputation comprised ankle-level or more proximal procedures. The single hospitalization with an unspecified level was omitted from the grouped analysis (overall *n* = 228; Al-Zahra *n* = 130; Kashani *n* = 98); grouped percentages were calculated using these denominators. Bold text indicates category group headings within the table.

**Table 3:** Age distribution across primary etiologic and amputation-level categories.

Category	*n*	Median (IQR), Years	Omnibus Comparison
Etiology: Trauma	113	31 (24–44)	H = 93.71; *p* < 0.001; ε^2^ = 0.398 (95% CI, 0.307–0.509)
Etiology: Dysvascular	66	65 (57–76)	
Etiology: Infection	24	47.5 (36.0–58.3)	
Etiology: Malignancy	4	56 (49.8–57.5)	
Etiology: Deformity	8	11 (7.3–18.5)	
Etiology: Other/indeterminate	14	51.5 (45.3–59.5)	
Level: Toe	81	33 (23–52)	H = 18.71; *p* = 0.0047; ε^2^ = 0.057 (95% CI, 0.016–0.156)
Level: Partial foot	11	49 (40–67)	
Level: Ankle	8	50 (26.3–61.5)	
Level: Below-knee	79	50 (31–64.5)	
Level: Knee disarticulation	17	64 (44–68)	
Level: Above-knee	30	49 (30–62)	
Level: Hip disarticulation	2	43.5 (37.3–49.8)	

IQR, interquartile range; CI, confidence interval. Age is reported as median (IQR). Omnibus age comparisons used the Kruskal–Wallis test; the *p* values shown are unadjusted. Effect-size intervals are 95% percentile bootstrap confidence intervals.

**Table 4:** Cross-tabulation of primary amputation etiology by anatomical amputation level among hospitalizations with a specified level.

Primary Etiology	Toe	Partial Foot	Ankle	Below-Knee	Knee Disart.	Above-Knee	Hip Disart.	Total *n*
Trauma-related	49 (43.8)	4 (3.6)	4 (3.6)	35 (31.2)	4 (3.6)	15 (13.4)	1 (0.9)	112
Dysvascular	12 (18.2)	6 (9.1)	3 (4.5)	27 (40.9)	8 (12.1)	10 (15.2)	0 (0.0)	66
Infection-related	6 (25.0)	1 (4.2)	0 (0.0)	10 (41.7)	3 (12.5)	4 (16.7)	0 (0.0)	24
Malignancy	0 (0.0)	0 (0.0)	0 (0.0)	2 (50.0)	0 (0.0)	1 (25.0)	1 (25.0)	4
Deformity	5 (62.5)	0 (0.0)	1 (12.5)	1 (12.5)	1 (12.5)	0 (0.0)	0 (0.0)	8
Other/indeterminate	9 (64.3)	0 (0.0)	0 (0.0)	4 (28.6)	1 (7.1)	0 (0.0)	0 (0.0)	14

Values are *n* (row %). One trauma-related hospitalization had an unspecified anatomical level and was excluded from this cross-tabulation (*n* = 228). Etiology and anatomical level were associated (Fisher–Freeman–Halton Monte Carlo raw *p* = 0.0143; Holm-adjusted *p* = 0.0286; bias-corrected Cramer’s V = 0.170, 95% CI 0.098–0.394). “Knee disart.” and “Hip disart.” denote knee and hip disarticulation, respectively; above-knee denotes transfemoral amputation.

**Table 5:** Exploratory multivariable logistic regression of major versus minor amputation.

Variable	Contrast/Reference	Adjusted OR (95% CI)	*p* Value
Age	Per 10-year increase	1.20 (1.04–1.39)	0.012
Sex	Female vs. male (reference)	2.65 (0.96–7.29)	0.060
Hospital	Al-Zahra vs. Kashani (reference)	4.25 (2.37–7.60)	<0.001

OR, odds ratio; CI, confidence interval. Complete cases with a specified amputation level: *n* = 228 (major, *n* = 136; minor, *n* = 92). Outcome: major versus minor amputation (reference). ORs are mutually adjusted for age, sex, and hospital; etiology was not included. Age linearity was assessed by comparing the linear term with a natural cubic spline (likelihood-ratio *p* = 0.949). Estimates are exploratory conditional associations and should not be interpreted as causal effects.

**Table 6:** Gregorian annual distribution of included hospitalizations and hospital-stratified temporal analyses. (**A**) Included hospitalizations by Gregorian calendar year. (**B**) Secondary temporal analyses within each hospital, restricted to complete calendar years 2015–2019.

**(A)**					
**Gregorian Year**		**Al-Zahra, *n***	**Kashani, *n***	**Total, *n***	
2014 *		18	8	26	
2015		24	9	33	
2016		36	28	64	
2017		34	14	48	
2018		8	16	24	
2019		9	23	32	
2020 *		1	1	2	
Total		130	99	229	
(**B**)					
**Hospital**	**Outcome**	**Test**		**Statistic**	***p* value**
Al-Zahra	Age	Kruskal–Wallis		H = 2.94	0.569
Al-Zahra	Primary etiology	Fisher–Freeman–Halton, Monte Carlo		-	0.910
Al-Zahra	Amputation level	Fisher–Freeman–Halton, Monte Carlo		-	0.181
Kashani	Age	Kruskal–Wallis		H = 3.13	0.536
Kashani	Primary etiology	Fisher–Freeman–Halton, Monte Carlo		-	0.486
Kashani	Amputation level	Fisher–Freeman–Halton, Monte Carlo		-	0.830

* Partial calendar year. Source admission dates were converted to Gregorian calendar dates. The observed admission date range was from 13 April 2014 to 18 March 2020; the intended extraction window and comprehensive hospital ascertainment could not be independently verified. Exploratory temporal comparisons were restricted to 2015–2019. Panel B analyses were secondary, and *p* values are unadjusted.

## Data Availability

The de-identified analytic data are not publicly available because of patient-confidentiality and ethical restrictions. They may be made available by the corresponding author upon reasonable request and subject to institutional and ethical approval. The statistical analysis script used for the revised analyses is supplied with the Supplementary Materials.

## Supplementary Materials

The following supporting information can be downloaded at https://www.mdpi.com/article/10.3390/healthcare14203518/s1. Figure S1: Flow of available extracted hospitalization records and analytic sample; Table S1: Complete code-based algorithm for classification of primary amputation etiology; Table S2: Pairwise age comparisons across primary etiologic categories using Dunn’s test with Holm adjustment; Table S3: Etiologic sensitivity analyses: combination of dysvascular/infection, exclusion of indeterminate records, and nested conservative nonspecific-code reclassification.; Table S4: Summary of the nine original exploratory inferential analyses; Table S5: Hospital-stratified analyses of age- and sex-related associations; Code S1: Statistical analysis script (Code_S1_Statistical_Analysis.py), including primary, sensitivity, hospital-stratified, adjusted, and temporal analyses.
