# Supplementary material for: Etiology and Anatomical Level of Lower-Limb Amputations in Two Iranian Referral Hospitals: A Retrospective Record-Based Study
Source: Healthcare (Basel). 2026 Oct 19;14(20):3518. doi: 10.3390/healthcare14203518 (PMC13704086; doi:10.3390/healthcare14203518)
Supplement: Supplementary file 1 [file healthcare-14-03518-s001.zip › healthcare-4590514-supplementary.pdf]

Supplementary Materials

Etiology and Anatomical Level of Lower-Limb Amputations in Two Iranian Referral Hospitals: A Retrospective Record-Based Study

Hossein Akbari Aghdam, Mehdi Motififard, Hasan Yazdekhasti, Fouad Rahimi, Mahsa Jamali and Mohammad Shahsavan*

Supplementary Figure S1, Tables S1–S5, and Code S1

Code S1. Statistical analysis script (Code_S1_Statistical_Analysis.py), including primary, sensitivity, hospital-stratified, adjusted, and temporal analyses. The source hospital datasets are not distributed because of patient-confidentiality and ethical restrictions; the script requires a de-identified analytic CSV to run.

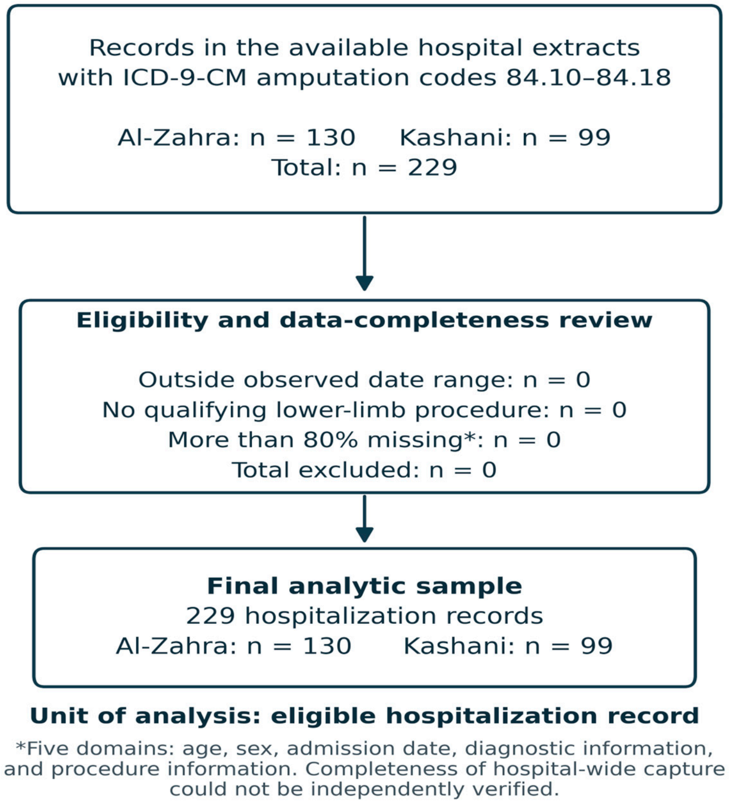

Figure S1. Flow of available extracted hospitalization records and analytic sample. The two hospital source extracts contained 229 hospitalization records with ICD-9-CM lower-limb amputation procedure codes 84.10-84.18 (Al-Zahra, n = 130; Kashani, n = 99). No records were excluded after eligibility and data-completeness review. The prespecified >80% missing-data criterion was operationalized across five core analytic domains: age, sex, admission date, diagnostic information, and procedure information. The unit of analysis was the eligible hospitalization record. The diagram documents eligibility within the available extracts; it does not establish comprehensive ascertainment of all eligible hospital amputations.

Table S1. Complete code-based algorithm for classification of primary amputation etiology.

Panel A. Exact observed diagnostic codes eligible to determine etiologic classification and operational hierarchy.

Etiologic category / role	Exact observed ICD-10 diagnostic codes	Operational rule and precedence
Trauma-related	S71.1; S72.0; S72.00; S72.1; S72.20; S72.30; S72.31; S72.40; S72.41; S72.71; S72.90; S72.91; S75.0; S76.4; S77.1; S78.1; S81.0; S81.9; S82.10; S82.11; S82.20; S82.21; S82.30; S82.40; S82.41; S82.50; S82.51; S82.71; S82.80; S82.81; S83.0; S84.0; S84.1; S85.0; S85.1; S85.5; S87.0; S87.8; S88.0; S88.1; S88.9; S91.0; S91.2; S91.3; S92.00; S92.10; S92.11; S92.20; S92.21; S92.30; S92.31; S92.40; S92.41; S92.5; S92.50; S92.51; S92.71; S93.1; S93.3; S93.5; S96.1; S96.7; S97.0; S97.1; S97.8; S98.0; S98.1; S98.2; S98.4; T04.3; T07; T13.4; T13.6; T93.0; T93.1; T93.2; T93.6; T93.8; T93.9; T95.3	A specific lower-limb injury, traumatic amputation, crush injury, fracture, vascular injury, or documented lower-limb injury sequela was considered a specific traumatic cause. If R02 or L97 preceded a specific lower-limb injury/sequela code, the specific traumatic code took precedence. Anatomically unrelated injury codes did not establish traumatic etiology. T07 is an unspecified multiple-injury code, not confirmation of a lower-limb injury. One T07-classified hospitalization lacked another explicit lower-limb injury diagnostic code; clinical verification was unavailable.
Dysvascular - vascular codes (including I99)	E10.5; E14.5; I73.1; I74.3; I99	Diabetes with peripheral circulatory complications or documented lower-limb peripheral vascular/arterial occlusive disease was classified as dysvascular when it was the principal or first specific etiologic diagnosis. I99 is an anatomically nonspecific circulatory diagnosis. Its use as an etiologic surrogate in two hospitalizations without a separate limb-specific vascular or ulcer/gangrene diagnosis was not confirmed by chart review and may cause misclassification.
Dysvascular - nonspecific manifestations	L97; R02	R02 (gangrene) and L97 (ulceration) were treated as nonspecific manifestations. They were classified as dysvascular only when no more specific competing etiologic code was identified in the ordered diagnostic fields.
Infection-related	M86.6; M86.67; M86.8; M86.86; M86.87; M86.9; M86.96; M86.97; T81.4; T84.6; T84.7; T87.4	Osteomyelitis or documented postoperative, stump, or internal-device infection was classified as infection-related when it was the principal or first specific etiologic diagnosis.
Malignancy	C40.2; C49.2; C76.5	A specific malignant neoplasm involving the lower limb was classified as malignancy when it was the principal or first specific etiologic diagnosis.
Deformity	Q66.0; Q69.2; Q69.9; Q72.8	A congenital/non-traumatic lower-limb deformity was classified as deformity when it was the principal or first specific etiologic diagnosis and no earlier specific competing cause was present.
Other/indeterminate	E78.3; F11.2; F17.2; J44.9; M34.9; M84.16; M87.87; T84.8; T86.8; T87.5; Z48.8; Z91.6	Residual category. Hospitalizations were retained as other/indeterminate when no diagnostic code in the ordered fields met a specific etiologic rule and classification would otherwise require assumption. The codes shown are the exact codes observed in hospitalizations ultimately retained in this category; they are not a single etiologic code set.

Exact hierarchy: the principal/first recorded diagnosis was evaluated first. If it identified a specific etiologic process, that category was assigned. If the first diagnosis was nonspecific or nonetiologic, subsequent diagnoses were reviewed in recorded order and the first specific anatomically relevant etiologic code determined classification. If no specific code was identified but R02 or L97 was present, the hospitalization was classified as dysvascular. If none of these rules yielded a defensible category, the hospitalization was retained as other/indeterminate. For Al-Zahra, the final principal diagnosis field preceded the additional final-diagnosis field. For Kashani, final-diagnosis fields 1-8 were evaluated in recorded order. Duplicate codes within a hospitalization were ignored after their first occurrence. Age, sex, hospital, and amputation level were not used to assign etiology.

Observed nonspecific-code assignments: four dysvascular hospitalizations depended on R02 and/or L97 fallback (no more-specific qualifying code under the prespecified hierarchy); four dysvascular hospitalizations were assigned on I99 (two without R02/L97 and two accompanied by R02); and one traumatic hospitalization depended on T07. All nine hospitalizations came from Al-Zahra Hospital. These diagnostic-basis categories are mutually exclusive. Reassignment to other/indeterminate in sensitivity analyses is an uncertainty test and not a new chart-validated clinical diagnosis.

Panel B. Complete observed diagnostic-code inventory not used as a specific etiologic determinant under the final algorithm.

Observed code list 1	Observed code list 2	Observed code list 3	Observed code list 4
A41.9	I89.9	N18.9	S52.40
C26.0	J44.9	R07.4	S52.50
D64.9	J90	R99	S52.7
E11.9	J95	S01.3	S52.70
E14.6	K92.2	S02.00	S60.9
E14.7	L08.9	S02.40	S62.61
E14.9	L89	S02.80	S63.0
E78.3	L90.5	S05.9	S63.1
F11.2	L92.8	S27.0	S67.8
F15.2	L92.9	S27.00	T79.6
F17.2	M14.6	S27.2	T81.3
F29	M21.6	S31.0	T81.7
F43.2	M21.67	S31.1	T82.8
F63.9	M21.97	S31.8	T84.8
G12.2	M25.67	S32.00	T86.8
G57.3	M34.9	S32.30	T87.5
G57.4	M84.15	S32.40	T87.6
I20.0	M84.16	S32.80	T88.8
I26.9	M87.26	S36.1	Z48.8
I48	M87.27	S37.2	Z86.7
I64	M87.37	S42.20	Z87.7
I69.4	M87.87	S42.30	Z91.6
I80.2	M87.97	S47	Z92.4
I80.3	N18.0	S52.00	Q69.0

The two source datasets contained 202 unique diagnostic codes. Panels A and B together provide the complete observed diagnostic-code inventory; Panel B lists codes that did not independently determine the final etiologic category under the predefined hierarchy.

Panel C. Worked classification examples observed in the source data.

Observed diagnostic combination	Final category	Application of hierarchy
R02 + T93.8	Trauma-related	R02 is nonspecific; the later specific lower-limb injury-sequela code T93.8 takes precedence.
L97 + R02	Dysvascular	Only nonspecific ulceration/gangrene manifestations were present; no more specific competing cause was documented.
E14.5 + M86.9	Dysvascular	E14.5 was the principal/first specific diagnosis; the later osteomyelitis code did not override the principal specific etiology.
M86.8 + E14.5	Infection-related	M86.8 was the principal/first specific diagnosis; the later diabetes-with-circulatory-complication code did not override it.
I74.3 + T81.4	Dysvascular	I74.3 was the principal/first specific diagnosis; the later postoperative-infection code did not override it.
T87.4 + E14.9	Infection-related	A specific stump-infection code was the principal diagnosis; uncomplicated diabetes did not determine etiology.

Panel D. Complete external-cause code inventory observed in the source datasets.

External-cause code	All hospitalizations, n	Trauma-classified, n	External-cause code	All hospitalizations, n	Trauma-classified, n
V03.1	3	3	V74.6	1	1
V03.19	1	1	V84.9	2	2
V06.1	1	1	W18.9	1	0
V22.0	1	1	W20	3	3
V22.4	4	4	W20.62	5	5
V22.9	1	1	W20.9	3	3
V23.4	17	17	W24.9	1	1
V23.49	2	2	W31	5	5
V23.5	2	2	W31.62	7	7
V23.9	1	1	W34	2	2
V24.4	3	3	W40	1	1
V24.5	1	1	W55.9	1	1
V24.59	1	1	W87	1	1
V27.4	1	1	Y34.9	1	1
V28.4	3	3	Y45.0	1	1
V29.4	1	1	Y83.0	1	0
V35.19	1	1	Y83.2	1	0
V43.6	1	1	Y83.5	4	1

External-cause code	All hospitalizations, n	Trauma-classified, n	External-cause code	All hospitalizations, n	Trauma-classified, n
V44.6	1	1	Y83.8	5	0
V47.6	1	1	Y85.0	1	1
V48.6	1	1	Y85.9	3	2
V63.6	1	1	Y86	8	8
V64.5	1	1	Y87.2	5	3
V67.6	1	1	Y88.1	1	1

External-cause codes were reviewed as supporting contextual/mechanism information and were not sufficient on their own to assign final primary etiology. External-cause coding was present in 98 of 113 trauma-related hospitalizations (86.7%). The trauma category was reconstructed from injury and injury-sequela coding. One hospitalization categorized as trauma involved an anatomically nonspecific T07 code without an additional limb-specific injury diagnosis; this classification was not clinically verified. Independent duplicate classification, inter-rater agreement, and validation against complete clinical charts were not available in the source project files and are therefore not claimed.

Table S2. Pairwise age comparisons across primary etiologic categories using Dunn's test with Holm adjustment.

Comparison	Dunn z	Raw p	Holm-adjusted p	Rank-biserial r (95% CI)
Trauma vs dysvascular	-8.635	<0.001	<0.001	-0.747 (-0.845 to -0.633)
Trauma vs infection	-3.160	0.0016	0.0173	-0.489 (-0.672 to -0.285)
Trauma vs malignancy	-1.449	0.1473	0.7364	-0.531 (-0.792 to -0.179)
Trauma vs deformity	2.671	0.0076	0.0681	0.779 (0.492 to 0.966)
Trauma vs other/indeterminate	-2.420	0.0155	0.1088	-0.482 (-0.709 to -0.221)
Dysvascular vs infection	2.633	0.0085	0.0681	0.474 (0.218 to 0.706)
Dysvascular vs malignancy	1.166	0.2436	0.9743	0.542 (0.235 to 0.818)
Dysvascular vs deformity	6.184	<0.001	<0.001	0.981 (0.939 to 1.000)
Dysvascular vs other/indeterminate	2.217	0.0266	0.1599	0.554 (0.331 to 0.751)
Infection vs malignancy	-0.050	0.9601	1.000	-0.063 (-0.625 to 0.563)
Infection vs deformity	4.133	<0.001	<0.001	0.964 (0.865 to 1.000)
Infection vs other/indeterminate	0.073	0.9414	1.000	-0.071 (-0.446 to 0.298)
Malignancy vs deformity	2.800	0.0051	0.0511	0.938 (0.625 to 1.000)
Malignancy vs other/indeterminate	0.091	0.9273	1.000	0.054 (-0.571 to 0.643)
Deformity vs other/indeterminate	-3.752	<0.001	0.0021	-0.964 (-1.000 to -0.839)

Dunn's pairwise comparisons were performed after the significant omnibus Kruskal-Wallis test. Holm adjustment was applied across all 15 pairwise comparisons. Rank-biserial correlations are presented with 95% percentile bootstrap confidence intervals based on 10,000 resamples. A negative rank-biserial correlation indicates that the first-listed etiologic group tended to be younger than the second-listed group.

Table S3. Etiologic sensitivity analyses: combination of dysvascular/infection, exclusion of indeterminate records, and nested conservative nonspecific-code reclassification.

Sensitivity analysis	n	Test	Statistic	Effect size (95% CI)	p value
Age across five sensitivity etiology categories	229	Kruskal-Wallis	H = 86.78	epsilon-squared = 0.370 (0.277-0.479)	<0.001
Hospital × sensitivity etiology	229	Fisher-Freeman-Halton Monte Carlo	-	Bias-corrected Cramer's V = 0.379 (0.285-0.487)	<0.001
Sensitivity etiology × amputation level	228	Fisher-Freeman-Halton Monte Carlo	-	Bias-corrected Cramer's V = 0.200 (0.103-0.441)	0.0034
Hospital × etiology after excluding other/indeterminate	215	Fisher–Freeman–Halton Monte Carlo	—	Bias-corrected Cramér's V = 0.293 (0.195–0.416)	<0.001
Reassign three I99-alone/T07: age × etiology	229	Kruskal–Wallis	H = 90.45	ε^2 = 0.383 (0.292–0.495)	<0.0001
Reassign three I99-alone/T07: Hospital × etiology	229	Fisher–Freeman–Halton Monte Carlo	—	Bias-corrected V = 0.328 (0.235–0.454)	<0.0001
Reassign three I99-alone/T07: Sex × etiology	229	Fisher–Freeman–Halton Monte Carlo	—	Bias-corrected V = 0.205 (0.064–0.400)	0.0093
Reassign three I99-alone/T07: Etiology × level	228	Fisher–Freeman–Halton Monte Carlo	—	Bias-corrected V = 0.162 (0.085–0.389)	0.0353
Reassign seven incl. R02/L97 fallback: age × etiology	229	Kruskal–Wallis	H = 88.63	ε^2 = 0.375 (0.288–0.486)	<0.0001
Reassign seven incl. R02/L97 fallback: Hospital × etiology	229	Fisher–Freeman–Halton Monte Carlo	—	Bias-corrected V = 0.281 (0.189–0.411)	0.00011
Reassign seven incl. R02/L97 fallback: Sex × etiology	229	Fisher–Freeman–Halton Monte Carlo	—	Bias-corrected V = 0.202 (0.059–0.400)	0.0098
Reassign seven incl. R02/L97 fallback: Etiology × level	228	Fisher–Freeman–Halton Monte Carlo	—	Bias-corrected V = 0.159 (0.082–0.389)	0.0522
Reassign nine incl. I99 with R02: age × etiology	229	Kruskal–Wallis	H = 86.82	ε^2 = 0.367 (0.278–0.478)	<0.0001
Reassign nine incl. I99 with R02: Hospital × etiology	229	Fisher–Freeman–Halton Monte Carlo	—	Bias-corrected V = 0.262 (0.173–0.393)	0.00036
Reassign nine incl. I99 with R02: Sex × etiology	229	Fisher–Freeman–Halton Monte Carlo	—	Bias-corrected V = 0.220 (0.076–0.419)	0.0056
Reassign nine incl. I99 with R02: Etiology × level	228	Fisher–Freeman–Halton Monte Carlo	—	Bias-corrected V = 0.160 (0.084–0.389)	0.0525

For this secondary sensitivity analysis, dysvascular (n = 66) and infection-related (n = 24) hospitalizations were combined into a single dysvascular/infection category (n = 90; Al-Zahra, n = 67; Kashani, n = 23), yielding five etiologic categories. The same statistical procedures used in the primary analyses were repeated. The level-based analysis excluded the single hospitalization with unspecified anatomical level (n = 228). These secondary p values were not included in the Holm family used for the original inferential analyses. A separate sensitivity analysis excluded 14 other/indeterminate hospitalizations (n = 215) when testing hospital-by-etiology association.

For the reviewer-requested diagnostic-code sensitivity analyses, the three most uncertain assignments comprised two I99-based dysvascular records without R02/L97 and one T07-based trauma record. Four additional dysvascular hospitalizations were based on the fallback R02/L97 rule. The seven-record sensitivity analysis reassigned all seven to other/indeterminate; the nine-record scenario additionally reassigned the two I99-with-R02 hospitalizations. No records were removed in these reclassification analyses (total n = 229; n = 228 for tests involving anatomical level). All nine flagged records originated at Al-Zahra Hospital. Monte Carlo p values used 200,000 fixed-margin resamples; confidence intervals for effect sizes used 10,000 percentile bootstrap resamples. These reviewer-requested secondary p values were unadjusted and were not included in the original Holm correction family. A p value above 0.05 for the etiology-by-level association was observed in the seven- and nine-record scenarios.

Table S4. Summary of the nine original exploratory inferential analyses.

Comparison	Statistical test	Test statistic	Effect size (95% CI)	Raw p	Holm-adjusted p
Age across etiologic categories	Kruskal-Wallis	H = 93.71; df = 5	ε^2 = 0.398 (0.307-0.509)	<0.001	<0.001
Age across amputation levels	Kruskal-Wallis	H = 18.71; df = 6	ε^2 = 0.057 (0.016-0.156)	0.0047	0.0188
Age by sex	Mann-Whitney U	U = 1946.0	Rank-biserial r = -0.366 (-0.572 to -0.142)	0.0011	0.0064
Age by hospital	Mann-Whitney U	U = 7483.0	Rank-biserial r = 0.163 (0.009-0.307)	0.0349	0.0349
Sex × primary etiology	Fisher-Freeman-Halton, Monte Carlo	-	Bias-corrected Cramér's V = 0.215 (0.073-0.411)	0.0065	0.0195
Sex × amputation level	Fisher-Freeman-Halton, Monte Carlo	-	Bias-corrected Cramér's V = 0.260 (0.117-0.457)	0.0032	0.0162
Etiology × amputation level	Fisher-Freeman-Halton, Monte Carlo	-	Bias-corrected Cramér's V = 0.170 (0.098-0.394)	0.0143	0.0286
Hospital × primary etiology	Fisher-Freeman-Halton, Monte Carlo	-	Bias-corrected Cramér's V = 0.374 (0.285-0.486)	<0.001	<0.001
Hospital × amputation level	Fisher-Freeman-Halton, Monte Carlo	-	Bias-corrected Cramér's V = 0.471 (0.374-0.592)	<0.001	<0.001

CI, confidence interval. The nine original inferential analyses were treated as one exploratory family and adjusted using the Holm procedure. These analyses are pooled and unadjusted for hospital unless hospital itself is the comparison variable; hospital-stratified secondary analyses are reported in Supplementary Table S5, and the major-versus-minor logistic model includes hospital as a covariate. Bias-corrected Cramer's V uses the finite-sample correction. Effect-size 95% CIs were obtained from 10,000 percentile bootstrap resamples. epsilon-squared = (H - k + 1)/(n - k).

Table S5. Hospital-stratified analyses of age- and sex-related associations.

Hospital	n analyzed	Association	Test	Statistic	Effect size (95% CI)	p value
Al-Zahra	130	Age × primary etiology	Kruskal-Wallis	H = 50.25	epsilon-squared = 0.370 (0.242-0.526)	<0.001
Kashani	99	Age × primary etiology	Kruskal-Wallis	H = 41.39	epsilon-squared = 0.391 (0.244-0.578)	<0.001
Al-Zahra	130	Age × sex	Mann-Whitney U	U = 600.0	Rank-biserial r = -0.455 (-0.694 to -0.190)	0.0013
Kashani	99	Age × sex	Mann-Whitney U	U = 388.5	Rank-biserial r = -0.197 (-0.559 to 0.203)	0.290
Al-Zahra	130	Age × amputation level	Kruskal-Wallis	H = 12.57	epsilon-squared = 0.053	0.050
Kashani	98	Age × amputation level	Kruskal-Wallis	H = 7.23	epsilon-squared = 0.024	0.204
Al-Zahra	130	Sex × primary etiology	Fisher-Freeman-Halton Monte Carlo	-	Bias-corrected Cramer's V = 0.323 (0.094-0.512)	0.005
Kashani	99	Sex × primary etiology	Fisher-Freeman-Halton Monte Carlo	-	Bias-corrected Cramer's V = 0.050 (0.000-0.450)	0.224
Al-Zahra	130	Sex × amputation level	Fisher-Freeman-Halton Monte Carlo	-	Bias-corrected Cramer's V = 0.214 (0.000-0.485)	0.067
Kashani	98	Sex × amputation level	Fisher-Freeman-Halton Monte Carlo	-	Bias-corrected Cramer's V = 0.238 (0.000-0.566)	0.104

These hospital-stratified analyses were secondary. Age comparisons used Kruskal–Wallis or Mann–Whitney U tests (ε^2 or rank-biserial r); categorical associations used Fisher–Freeman–Halton Monte Carlo tests (bias-corrected Cramér's V). All effect-size 95% CIs used 10,000 percentile bootstrap resamples. Level-based analyses excluded one Kashani hospitalization with unspecified level (Kashani n = 98). Sparse categories precluded reliable six-category etiologic regression; the separate major/minor logistic model adjusted for age, sex, and hospital.
